# Examining colorectal cancer screening uptake and health provider recommendations among underserved middle aged and older African Americans

**DOI:** 10.34172/hpp.2022.52

**Published:** 2022-12-31

**Authors:** Sharon Cobb, Tavonia Ekwegh, Edward Adinkrah, Hoorolnesa Ameli, Attallah Dillard, Lucy W. Kibe, Mohsen Bazargan

**Affiliations:** ^1^Mervyn M. Dymally School of Nursing, Charles R. Drew University of Medicine and Science (CDU), CA, USA; ^2^Department of Public Health, College of Science & Health, CDU, CA, USA; ^3^Mellie’s Bank Hospital, Tehran, Iran; ^4^Physician Assistant Program, College of Science & Health, CDU, CA, USA; ^5^Department of Family Medicine, College of Medicine, CDU, CA, USA; ^6^Department of Family Medicine, David Geffen School of Medicine at UCLA, CA, USA

**Keywords:** Mass screening, Early detection of cancer, Colonoscopy, Health maintenance organizations, Black or African American, Depression, Quality of health care, Aged

## Abstract

**Background:** The purpose of this study is to determine whether underserved middle-aged and older African Americans are receiving a colorectal cancer (CRC) screening test (sigmoidoscopy or colonoscopy) and if recommended by their provider. Additionally, we examined correlates of both provider recommendation and uptake of CRC screening.

**Methods:** Seven hundred forty African American individuals, aged 55 and older, participated in this local community cross-sectional survey. We used a multivariate technique of logistic regression.

**Results:** One out of three participants reported that they never received a sigmoidoscopy or colonoscopy for CRC screening. More than 31% indicted that their providers never suggested CRC testing. However, participants who indicated that their providers recommended sigmoidoscopy/colonoscopy were almost 49 times (odds ratio [OR]: 48.9, 95% confidence interval [CI]: 29.5–81.2) more likely to obtain it compared to their counterparts who were not advised to have these procedures. Our data suggest that African American men were significantly less likely than women to receive recommendations from their providers (OR: 0.70, 95% CI: 0.50-0.91). Furthermore, controlling for other variables, the following factors: 1) living arrangement (OR: 1.44, 95% CI: 1.02–2.04), 2) health maintenance organization (HMO) membership (OR: 1.84, 95% CI: 1.28–2.67), 3) number of providers (OR: 1.15, 95% CI: 1.01–1.32), 4) satisfaction with access to and quality of care (OR: 1.24, 95% CI: 1.03–1.51), 5) depressive symptoms (OR: 0.92, 95% CI: 0.86–0.98), and 6) gastrointestinal conditions (OR: 1.73, 95% CI: 1.16–2.58) were associated with obtaining a sigmoidoscopy or colonoscopy test.

**Conclusion:** Our findings suggest that the absence of a provider recommendation is the primary barrier preventing underserved older African Americans from obtaining CRC screening. In addition, our data revealed significant association between obtaining CRC screening and some of the predisposing characteristics of participants, satisfaction with access to and quality of care, and physical and mental health. These findings are consistent with this notion that disparities in health care for African Americans can be traced back to four primary factors: patients, healthcare providers, the healthcare system, and society as a whole, and emphasize the need for establishing theory-driven, culturally-sensitive, and cost-effective CRC screening interventions that recognize and address the constraints to cancer screening experienced by this segment of population.

## Introduction

 Colorectal cancer (CRC) is the fourth frequent disease recognized in the United States and the third cancer-related cause of death despite the greater promotion of screening guidelines.^1,2^ Around 151 000 people were diagnosed with CRC in 2022 and at least 52 580 will die from colon cancer in the U.S.^1^ Guidelines for recommended CRC screening include visual examination and fecal immunochemical test, that can lower CRC rates and mortality by identifying polyps and tumors earlier than usual.^3-7^ Evidence shows that visual examination tests, including colonoscopy and sigmoidoscopy tests, are more preferred and effective in reducingservice CRC incidence compared to stool-based tests.^8^ Latest recommendations for colon cancer detection from the American Cancer Society (ACS) recommend screening begin at age 45 for adults of average risk, and at younger ages below 45 for individuals with higher CRC risk.^7^ Additionally, the US Preventive Services Taskforce published updated CRC guidelines in August 2021 that recommend screening start at 45 years.^2,9,10^

 Middle-aged and older adults are highly encouraged to participate in CRC screening due to age being recognized in the ranks of the most prominent CRC risk variables. Even though recent evidence reports CRC increasing in younger ages, about 94% of all new instances of CRC are found in people aged 45 and up.^9^ Among older adults, CRC incidence doubles every decade of life from age 40 to 80.^11^ Among adults age 76 and older, CRC screening is recommended to continue in accordance with individual patient priorities, current health, and past screening results^7^, due to a possible increased risk of adverse events associated with older age and comorbidities. However, some studies have refuted that there is no established evidence that CRC screening should stop at a specific age due to the heterogeneity of older adults.^12,13^ Singhal and colleagues found that although older adults, specifically African Americans and Hispanics, may have a previously normal colonoscopy, screening in ages 75 or older can still detect advanced CRC (19%).^14^ About 5,200 middle-aged and older persons found a reduction of 65% in right-colon cancers and 75% in left-colon/rectal cancers after participating in CRC screening, specifically colonoscopy.^15^

 Among African Americans, rates of CRC are dramatically higher compared to non-Hispanic Whites.^16,17^ The ACS revealed that rates of occurrence and Death of CRC in African Americans were almost 20 and 40% greater among non-Hispanic whites, respectively.^3^ Research on racial disparities and stage-specific CRC uncovered that despite the CRC mortality rate decreasing over the years, the decline has been greater for non-Hispanic Whites at every stage of diagnosis.^18^ Another study focusing on screening outcomes among older African Americans, ages 75 and up similarly found higher rates of CRC among high-risk individuals.^19^ Overall, African Americans have lower rates of cancer screening, including participation in colonoscopy tests.^20^ Screening prevalence varies by race, with non-Hispanic Whites (63%) having higher CRC screening rates than African-Americans (59%).^6^ Sixty-four percent of men and 70% of women followed CRC screening standards in a recent study of 420 underprivileged African Americans in South Los Angeles.^21^ The disparity in CRC incidence among African-Americans and non-Hispanic Whites is 42% attributable to racial inequalities in screening.^22^

 Additionally, African Americans had a lower rate of follow-up visits with their doctors than non-Hispanic Whites regarding abnormal findings of CRC screenings, furthering racial disparities.^23^ Reviewing current literature, Rutter and colleagues found that lower rates of CRC screening are principally responsible for the increased rates of CRC occurrence across African American patients.^24^ Recent research by Barbieri indicates that the increased colorectal death rate among African Americans may be attributable to a lack of availability to and a lesser usage of high-quality colon cancer screening methodologies like colonoscopy (which has been shown to have larger ability to detect CRC abnormalities than computed tomography colonography imaging, faecal immunochemical testing, and stool guaiac testing).^25^

 In the United States, African Americans experience racial and ethnic disparities in CRC occurrence and death, which has been acknowledged in the latest clinical guidelines for CRC screening. The American College of Gastroenterology has explicitly highlighted the importance of developing community-outreach programs and initiatives intending to promote CRC testing amongst African Americans.^26^ Study findings suggest addressing the underutilization of CRC screening as a critical component to improving CRC outcomes, including CRC prevention, for African Americans.^18,27,28^ Despite an understanding of the implications of increasing CRC screening, there remains a limited and lack of consistency in understanding of the factors that influence the suggestion of a healthcare provider and uptake of CRC preventive services, specifically sigmoidoscopy and colonoscopy tests, among underserved minority adults. In order to design effective measures taken by local groups to improve CRC screening rates among underserved African American older adults, additional descriptive and correlational studies are needed. The goal of the current research was to establish a set of risk and protective factors with receipt of providers’ recommendation and obtaining sigmoidoscopy/colonoscopy tests among underserved middle-aged and older African Americans in South Los Angeles, one of the most populated, underserved and under-resourced areas of Los Angeles County. This study sought to conceptualize the contextual factors at the local and community-level to elucidate the impact of CRC screening uptake among minority and under-resourced communities. Sociodemographic characteristics, living arrangements, financial strain, availability of medical care, accessibility and quality of medical care, physical co-morbidity, mental health status, and providers’ recommendation were among the independent variables employed to explain the variation of CRC screening.

## Materials and Methods

###  Design and setting

 This study was implemented via face-to-face interviews with 740 community-dwelling older African Americans residents in South Los Angeles, California. Study participants were asked to provide information on demographic factors, socioeconomic status (SES), status of physical and psychological health, as well as health care access and usage. This study is part of a larger collective project that examined the health status and wellness outcomes of older African Americans residing in a region where resources are scarce.^29-32^ Details on the survey instrument, data collection methods, and study setting has been described in previous studies.^29-35^

###  Samples and sampling

 More than a million people live in Service Planning Area 6 (SPA 6) of Los Angeles country, and some of those people took part in the study. The United States government has identified this region as both a medically disadvantaged region and a healthcare professional deficit area.^36^ SPA 6 includes the regional area of South Los Angeles. Compared to the rest of Los Angeles County, SPA 6 residents suffer disproportionately from a variety of health problems. When taking into account factors such as age, the death rate from CRC in South and West Los Angeles was 16.0 and 9.4 per 100 000, correspondingly.^37^

###  Measurements

*Providers’ CRC recommendation* was assessed by self-report. Participants were asked whether their healthcare provider (e.g., physician, nurse practitioner) recommended that they should have a sigmoidoscopy or colonoscopy.

*CRC testing*. In this study, participants were questioned about their prior experiences with sigmoidoscopy or colonoscopy test. To ensure comprehension of the question, the research team explained the process of a colonoscopy and sigmoidoscopy. If participants indicated that they received a colonoscopy and/or sigmoidoscopy, they were asked to report the year the procedure occurred. This pair of factors was formally defined as a yes/no category.

*Sociodemographic covariates*. These factors were used as covariates: sexual preference, age, education level, and housing situation. Years of schooling were used as an interval variable to measure educational achievement; higher scores indicated more years of schooling.

*Financial strain*.Participants were asked the following questions, “How often in the past year have you been unable to: 1) provide your family with enough food, 2) provide your family with enough clothing, 3) pay your rent or mortgage, 4) pay your utility costs, and 5) earn a living? These items were graded on a five-point scale, from 1 (never) to 5 (always), and the average of these five scores was used to determine the overall “financial hardship” score. More points meant that participants were in more severe financial straits. The internal consistency of this index was high (Cronbach’s Alpha: 0.93).

*Satisfaction with access to and quality of medical care* was assessed by asking participants three items that measured their level of access to and satisfaction with medical cares. Each item was measured on a scale of 1 to 5 (low = 1 to high quality/access). The average of these three scores was used, where a higher score indicated of a higher level of satisfaction with quality access to and quality of medical care.^34^ The value for Cronbach’s Alpha for this 3-item scale was α = 0.82).

*Health maintenance organization (HMO) membership* was examined by asking participants to provide their insurance carrier or the location where they receive medical care.

*Number of providers* was analyzed by asking participants to list the number of healthcare providers that they have received care from within the past 12 months. This variable was calculated as a continuous variable. Inspecting all medications’ containers, we documented the names and other information of all prescribers that had provided care to each participant.

*Major chronic and gastrointestinal conditions.* Participants were queried about the presence or absence of seven diseases or disorders: hypertension, diabetes mellitus, cardiovascular disease, stroke, cancer, and gastrointestinal problems. (i.e., Crohn’s disease, diverticulosis).

*Depression-related symptoms*. For this study, we used a 15-item survey called the Geriatric Depression Scale (GDS) to assess depression symptoms with a “yes” or “no” answer.^38^ The GDS is calculated using a summary score with a range between 0 and 15. Higher scores indicates a greater severity of depression. When it comes to measuring symptoms of depression in older persons in public, acute, and long-term care settings, this GDS has significant validity and reliability.^39^

###  Data analysis

 We divided our research into three phases. In the first phase, we conducted a descriptive evaluation of the entire sample, detailing the distributions of continuous measures and the percentages and frequencies of categorical ones. Our next stage included chi-square and independent t-tests to compare participants based on receiving a CRC screening recommendation and obtaining a sigmoidoscopy/colonoscopy test. The third stage centered on logistic regression to estimate the independent correlates of receiving a recommendation for sigmoidoscopy/colonoscopy by providers. We tested two models for examining the predictors of obtaining sigmoidoscopy/colonoscopy. The first multivariable logistic regression model include all independent variables except the providers’ recommendation, and the second Model included all variables in the first model and the providers’ recommendation. Missing data was observed in less than 5% of all variables. This study did not impute data, which were missing. In this study, significance was defined as a p-value of 0.05 or lower.

## Results

###  Descriptive characteristics

 Statistics on the study population is provided in Table 1. In total, 740 African American adults surveyed for this study were age 55 years and older (mean, 71.7 ± 8.3).Sixty-four percent or more of the participants were female, and around half of the women said they are living alone. At least 35% of the people in this sample had a bachelor’s degree or above, while 25% had only completed between zero and eleven years of schooling (i.e., below the level of a high school diploma). Additionally, more than a third of respondents (35%) said they use their HMO insurance for medical treatment. Table 2 shows that one out of three older African American participants had never been screened for CRC using a sigmoidoscopy or colonoscopy test. At least 30% of participants indicated that their providers had never recommended sigmoidoscopy or colonoscopy screening. Only one in five patients who reported that their doctor had suggested a sigmoidoscopy or colonoscopy screening had never obtained the CRC screening.

**Table 1:** Characteristics of Underserved African American Adults (n = 740)

	**Number**	**Percent**
Gender		
Male	266	35.9
Female	474	64.1
Age		
55-64	120	16.2
65-74	360	48.6
≥ 75	260	35.1
Education		
No high school diploma	183	24.7
High school diploma	265	35.8
Some college or college degree	292	39.5
Living alone		
No	294	39.7
Yes	446	60.3
Enrollment in the HMO		
No	478	64.6
Yes	262	35.4
Chronic gastrointestinal diseases		
No	506	68.6
Yes	232	31.4
		**Mean±SD**
Education attainment (1–16)		12.7 ± 2.24
Financial strains (1–5)		1.8 ± 1.13
Satisfaction with access to, availability, & quality of medical care (1: Low to 5: High)		3.4 ± 0.90
Number of providers (1–10)		2.19 ± 1.34
Depressive symptoms (0-15)		2.5 ± 2.77
Major chronic conditions (1–5)		2.1 ± 0.86

HMO, Health Maintenance Organization.

**Table 2:** Bi-variates correlates of colorectal cancer screening and independent variables among underserved African American Adults (n = 740)

**Independent variables**	**CRC Screening Recommended**		***P*** ** value**	**CRC Screening Performed**		***P*** ** value**
	**No** **N (%) or (X±SD)**	**Yes** **N (%) or (X±SD)**		**No** **N (%) or (X±SD)**	**Yes** **N (%) or (X±SD)**	
Gender			0.004			0.086
Male	101 (38)	165 (62)		99 (37)	167 (63)	
Female	132 (28)	342 (72)		147 (41)	327 (69)	
Age (55–96)	(71.2 ± 2.4)	(71.9 ± 7.8)	0.258	(70.3 ± 9.1)	(72.4 ± 7.9)	0.001
Education (1-15)	(12.7 ± 2.2)	(12.8 ± 2.3)	0.845	(12.8 ± 2.1)	(12.7 ± 2.3)	0.812
Financial stress (1: Low to 5: High)	(1.9 ± 1.2)	(1.8 ± 1.1)	0.312	(1.9 ± 1.1)	(1.8 ± 1.1)	0.086
Living alone			0.173			0.006
No	101 (34)	169 (66)		108 (37)	186 (63)	
Yes	132 (30)	314 (70)		138 (31)	308 (69)	
Access, availability & quality of medical care (1: Low to 3: High)	(3.3 ± 0.9)	(3.4 ± 0.9)	0.103	(3.2 ± 0.84)	(3.4 ± 0.92)	0.006
HMO membership			0.002			0.001
No	169 (35)	309 (65)		179 (37)	299 (63)	
Yes	64 (24)	198 (76)		67 (26)	195 (74)	
Number of providers (1 to 10)	(2.0 ± 1.2)	(2.3 ± 1.4)	0.004	(2.0 ± 1.2)	(2.3 ± 1.4)	0.013
Chronic gastrointestinal diseases			0.003			0.016
No	177 (35)	329 (65)		183 (36)	323 (64)	
Yes	56 (24)	176 (76)		63 (27)	169 (73)	
Number of chronic conditions (1-5)	(2.0 ± 0.9)	(2.1 ± 0.9)	0.902	(2.0 ± 0.86)	(2.1 ± 0.9)	0.548
Depressive symptoms (1-15)	(2.7 ± 2.9)	(2.3 ± 2.7)	0.176	(2.9 ± 3.1)	(2.3 ± 2.6)	0.002

Abbreviation: CRC, colorectal cancer; HMO, health maintenance organization.

###  Bi-variate analysis

Table 2 shows bivariate associations between receiving a CRC recommendation, obtaining sigmoidoscopy/colonoscopy, and other relevant variables. Columns 2-4 of this table reports that receiving a CRC recommendation was associated with gender, use of HMO, number of providers, and being diagnosed with chronic gastrointestinal (GI) disorders. Women, those who received their medical care from an HMO, had higher number of providers, and those who reported GI conditions were more likely to indicate that their providers recommended CRC screening using sigmoidoscopy/colonoscopy. Columns 5-7 of Table 2 reports bivariate correlations between obtaining sigmoidoscopy/colonoscopy and other relevant variables. Participants who obtained a sigmoidoscopy/colonoscopy test were older, lived alone, reported a greater degree of contentment with the availability and effectiveness of medical care, received their medical care from an HMO, had higher number of providers, were diagnosed with GI conditions, and had received a CRC recommendation from their providers.

###  Multivariate logistic regression

 Several logistic regressions were performed to correlates of CRC screening using sigmoidoscopy or/and colonoscopy and obtaining a CRC recommendation. Columns 2-4 of Table 3 reports correlates of receiving a CRC recommendation. Three out of four variables that demonstrated a strong correlation with a CRC recommendation at the bivariate level remained significant correlates of receiving CRC screening with sigmoidoscopy/colonoscopy. After controlling for all relevant variables, women (odds ratio [OR]= 1.43; 95% confidence interval [CI]:1.1–2.02), participants who had been diagnosed with GI conditions (OR = 1.83; 95% CI: 1.21–2.73), had received medical care from an HMO (OR = 1.53; 95% CI: 1.06–2.20), and those with a higher number of providers (OR = 1.17; 95% CI: 1.02–1.35) were more likely to have received a recommendation for CRC screening by sigmoidoscopy/colonoscopy from their provider.

**Table 3:** Multivariate logistic regression between independent variables and colorectal cancer recommendation and screening among underserved African American Adults (n = 740)

**Independent variables**	**CRC ** **recommendations**			**CRC screening** **Model 1**			**CRC screening ** **Model 2**		
	**OR**	**95% CI**	***P*** ** value**	**OR**	**95% CI**	***P*** ** value**	**OR**	**95% CI**	***P*** ** value**
Gender		1.1–2.02	0.045	1.01	0.71–1.43	0.965	0.62	0.37–1.02	0.063
Male	1.43								
Female	1.00
Age (55-96)	1.01	0.98–1.02	0.983	1.02	0.99–1.04	0.220	1.03	0.99–1.06	0.106
Education (1-15)	0.97	0.90–1.05	0.508	0.99	0.91–1.07	0.771	1.01	0.91 - 1.12	0.878
Living alone		0.88–1.76	0.213	1.44	1.02–2.04	0.037	1.55	0.95–2.55	0.083
No	1.25								
Yes	1.00
Financial stress (1: Low to 5: High)	0.99	0.84–1.19	0.984	1.01	0.84–1.12	0.979	1.01	0.78–1.29	0.998
Access, availability & quality of medical care (1: Low to 3: High)	1.18	0.97–1.35	0.099	1.24	1.03–1.51	0.025	1.24	0.94–1.62	0.123
HMO Membership		1.06–2.20	0.025	1.84	1.28–2.67	0.001	1.84	1.10–3.09	0.020
No	1.53								
Yes	1.00
Number of providers (1 to 10)	1.17	1.02–1.35	0.026	1.15	1.01–1.32	0.048	1.06	0.88–1.27	0.574
Chronic Gastrointestinal Diseases		1.21–2.73	0.004	1.73	1.16–2.58	0.007	1.32	0.75–2.33	0.331
No	1.82								
Yes	1.00
Number of major chronic conditions (1-5)	0.93	0.76–1.12	0.473	097	0.79–1.18	0.757	1.03	0.77–1.37	0.845
Depression symptoms (1-15)	0.95	0.89–1.02	0.143	0.92	0.86–0.98	0.011	0.89	0.81–0.98	0.024
Provider Recommended CRC		N/A			N/A		48.9	29.5–81.2	0.001
No									
Yes

Abbreviation: CRC, colorectal cancer; HMO, health maintenance organization.

 Columns 5-7 of Table 3 (Model 1) report correlates of obtaining sigmoidoscopy/colonoscopy with all independent variables except recommendation from providers. This logistic regression shows that six variables were associated with obtaining sigmoidoscopy/colonoscopy within the last 5 and 10 years, respectively. Participants who did not live alone (OR = 1.44; 95% CI: 1.02–2.04), those with greater contentment regarding medical care’s accessibility, availability, and effectiveness (OR = 1.24; 95% CI: 1.03–1.51), those who received their medical care from an HMO (OR = 1.84; 95% CI: 1.28–2.67), those with a higher number of providers (OR = 1.15; 95% CI: 1.01–1.32), those who have been diagnosed with GI conditions (OR = 1.73; 95% CI: 1.16–2.58), and those with lower number of depressive symptoms (OR = 0.92; 95% CI: 0.86–0.98) were more likely to be among those who obtained CRC screening using sigmoidoscopy/colonoscopy.

 Columns 8-10 of Table 3 (Model 2) report correlates of obtaining sigmoidoscopy/colonoscopy with all independent variables, including CRC recommendation from providers in the equation. This model shows that controlling for all other variables, in addition to providers’ recommendation, only HMO status and depressive symptoms remain significant predictors of obtaining CRC screening. Notably, while controlling for all other variables, participants who indicated that their providers recommended sigmoidoscopy/colonoscopy were almost 49 times (OR = 48.9; 95% CI: 29.5–81.2) more likely to obtain it than their counterparts who were not advised to have these procedures.

## Discussion

 This study identified factors associated with receiving providers’ CRC recommendations and obtaining a sigmoidoscopy/colonoscopy test within the population of older African Americans in South Central Los Angeles who are not receiving adequate care. Controlling for all relevant variables, multivariate logistic regression showed that gender, HMO membership, number of providers, and being diagnosed with gastrointestinal disorders were associated with receipt of providers’ recommendation for CRC screening using sigmoidoscopy/colonoscopy. CRC screening was more likely among participants who: (1) did not live alone, (2) received care from an HMO, (3) were satisfied with healthcare quality and accessibility, (4) had a higher number of providers, (5) had a lower number of depressive symptoms, and (6) had reported GI conditions. Individuals who said their doctors had encouraged them to get a sigmoidoscopy or colonoscopy were more likely to be screened.

 In our study of underprivileged older African Americans, enrollment in an HMO was found to be a significant predictor of both CRC screening adoption and CRC suggestion from clinicians. Multivariate logistic regression models show that even after all other relevant variables are controlled for; HMO insurance status remained a significant factor for obtaining sigmoidoscopy and/or colonoscopy within the last 5 and 10 years, respectively. HMO membership can be very beneficial, which may be attributed to intensive case management and prevention programs that are considered essential services in HMOs compared to non-HMOs.^40-45^ Mammography, the clinical breast exam, and the Pap smear are just a few of the cancer diagnostic tests that have been shown to rise in frequency along with the percentage of the population covered by HMOs in a given area.^46^ Analyzing individual and neighborhood data on 20,626 adult respondents 50 years and older from the 2005 California Health Interview Survey (CHIS), Shariff-Marco and colleagues documented that individual-level factors, including race and ethnicity, primary care shortage, and county-level HMO penetration, were associated with CRC screening using flexible sigmoidoscopy.^47^ Expansion of insurance coverage of CRC screening, specifically colonoscopy, can lead to increased use and higher probability of being diagnosed at an earlier cancer stage, as witnessed following Medicare reimbursement expansion.^48^ As a whole, due to accessible and efficient CRC screening, HMO participants are more likely to receive an early diagnosis of CRC.^49^

 In our study, we found that among the underserved, African American older adults who made up our sample, having multiple providers remains one of the determinants of CRC screening with sigmoidoscopy/colonoscopy, in addition to provider recommendation. Increasing people’s ability to get medical treatment has been explored to reduce racial disparities in CRC screening, but access alone might not be enough.^50^ Several studies have found that recommendations from healthcare provider (e.g. physician, Physician Assistant/Associate, nurse practitioner) to be a strong predictor in patient adherence with CRC screening.^51-56^ Seventy-six percent of 4283 older persons, as reported by Laiyemo et al, discussed CRC testing with their healthcare practitioner, which was likewise correlated with adherence to CRC screening standards.^57^ A perception that health care decisions should mostly be left to the physician rather than patients was found to be a strong predictor of a patient’s preparedness to be screened for CRC.^55^ Our findings are consistent with those of a recent systematic analysis of the literature, which found that the absence of a physician suggestion is the primary barrier preventing African Americans from obtaining CRC screening.^58^ However, despite the effectiveness of healthcare provider recommendations, some studies have found that CRC recommendations are not being given consistently.^51,54,59^

 This study’s findings showed that patients with gastrointestinal issues were more likely to have their doctors recommend CRC screening and go through with the procedure. Adults with GI conditions (i.e. inflammatory bowel disease, colitis) have a higher prevalence of CRC when compared to the general population.^60-62^ People with GI issues should have colonoscopies more frequently than routine screening and at shorter intervals than indicated by the guidelines.^63^ Friedman and colleagues reported that patients with colitis who remember discussions with their provider were more likely to have a positive outlook and participate in routin colonoscopies at appropriate intervals.^64^ Meta-analysis including 7199 studies found that colonoscopies reduce CRC and CRC-related mortality in those with gastrointestinal disorders.^65^ The effectiveness of CRC screening within minority adults with GI problems, particularly African Americans, needs more investigation.

 Our study also revealed that the availability of providers and approval of the medical services received and their quality remain significant correlates for CRC screening among this population. It is important to recognize that our study participants identify as a minority and reside in a low-income neighborhood/region within Los Angeles County, that is documented as a medically underserved area by the Health Resources and Services Administration (HRSA) of the federal government.^36^ A recent study conducted by Buehler and colleagues revealed that controlling for demographic and health care coverage, individuals in racially isolated communities had a 10% higher risk of not being screened for CRC than those of less isolated communities.^66^ Most African Americans receive care and medical services from non-African American (e.g. White) healthcare providers, which may reduce their satisfaction and rate of participation in preventive health activities due to medical mistrust and fear.^67,68^ A recent study by Alsan and colleagues found that after randomly assigning 1300 African Americans to African American or non-African American healthcare providers, African American patients reported that they had higher quality of care with African American doctors, with 34% receiving more preventive services.^69^ Social determinants of health and healthcare system challenges contribute to racial disparities observed in CRC. Moreover, the challenges of institutional and structural racism and cultural bias shape the access of African American communities to critical resources, including but not limited to CRC screening.

 Our sample of underprivileged older African Americans found that individuals with more depressive symptoms were less likely to follow their doctors’ recommendations to receive CRC screening. There is already an established relationship between depression and colon cancer.^70^ However, African Americans are less likely to seek help for depression, despite the fact that it is more disabling and widespread in this population.^71,72^ Additionally, it is well documented that depressive symptoms significantly reduce adherence to treatment and drug regimens.^73^ According to a recent study, depressed older persons utilize health care more frequently than the general population, but they do not take advantage of preventative interventions.^74^ Self-reported depressive symptoms were related to decreased rates of mammography screening, but not to CRC screening, in a large national data set from the United States among a healthy and self-motivated population of women.^75^ Therefore, screening for and treating depression is essential in providing preventative care for African American individuals of all ages.

 Our analyses also showed that living with others was associated with obtaining a colonoscopy or sigmoidoscopy. Social isolation and loneliness can influence cancer staging at diagnosis and survival,^76^ leading to increased cancer mortality risk..^77^ Our finding that African Americans who live with others may be more likely to obtain CRC screening is encouraging.

 It is important to note that our study population included underserved older African Americans, who may experience multiple barriers in obtaining cancer screenings, including distrust in the healthcare system.^78^ CRC has a greater prevalence and death rate in African Americans compared to Whites, which exacerbates the problem. Due to right-sided colon cancers being more common in African Americans, the first CRC screening test they should be referred for is a colonoscopy compared to other tests.^79^ Additionally, there is a sharp increase in middle-aged African Americans presenting with higher suspicions of CRC, which may include unusual symptoms, which should prompt providers to recommend CRC screening at younger ages below the guidelines.^80,81^ African Americans with multiple chronic conditions should be prompted by providers for timely CRC screening since it is linked poorer outcomes, including death, in this population.^82,83^ Due to African Americans being less likely to get screened for CRC, appropriate CRC education and culturally tailored strategies are needed to increase awareness of the value of CRC screenings.^84^

###  Implications

 Underserved populations, including minorities, may have lower awareness and access to CRC screening, which may serve as a barrier to community-based healthcare.^85^ It is important to note that existing health disparities within the African American population are related to patient, physician, system, and societal barriers.^86^ Therefore, community-based studies are needed to increase provider recommendations within performance safety-net clinics, private health systems, and HMOs in underserved and under-resourced communities for invasive and relatively expensive preventive cancer screenings, including sigmoidoscopy/colonoscopy. Further research centered on theory-driven, culturally sensitive, and cost-effective CRC screening interventions among the African American population is critically needed. These studies should focus on innovative education and screening-based interventions, such as mobile screening units and faith-based strategies that integrate sociocultural factors. Additionally, community health professionals, such as parish and public health nurses, can be utilized to advocate for the importance of CRC screenings and reduce medical mistrust among underserved and minority populations through tailored navigation and care. Furthermore, our study documented that African American men are less likely to receive recommendation for screening from their health care providers. Finding effective strategies to increase CRC screening recommendations and performance among African men requires more study.^87^

 Disparities in health care utilization resulting in lower screening and follow-up among African Americans could be because of a miscommunication between patient and doctor.^23^ Katz and colleagues found that among African Americans, individuals who said they had difficulty communicating with their doctors were less likely to have completed CRC screening within the recommended guidelines (OR = 2.8).^88^ Another recent study comparing African Americans and Whites reported that African Americans are more likely than their Whites to seek information for CRC screening using flexible sigmoidoscopy or colonoscopy information than their white Americans.^89^ However, it is not clear why providers are not effectively engaging their African Americans’ patients in CRC screening. This may be attributed to healthcare providers not having an understanding of CRC risks and phases of disease progression to appropriately recommend screening strategies.^90^ Two studies also found that a lack of shared decision-making about the CRC screening type reduced screening adherence.^91,92^ Considering the effect of racial disparities on CRC screening, interventional studies to encourage and educate healthcare practitioners in underprivileged areas to follow national screening guidelines are warranted.

 Despite CRC being one of the most preventative cancers due to advanced screening tests, there are still lower participation rates, especially in medical underserved communities. However, various strategies and policies can be implemented to increase uptake. From 2003 to 2014, New York City increased the screening colonoscopy rate from 42% to nearly 70% using various methods. However, the U.S. Surgeon General has ordered all clinics and ambulatory care facilities to postpone elective medical treatments and operations, including colonoscopies, due to the outbreak of the coronavirus 2019 (COVID-19) pandemic.^93^ This action is necessary for COVID-19 risk reduction, but it will potentially increase CRC disparities for underserved populations, including African Americans. Due to COVID-19 related pauses in medical care, culturally appropriate messaging and public campaigns should be advocated that will continue to encourage adults to meet with their provider and to discuss alternative strategies for timely screenings.

 There are a number of drawbacks to this study’s validity, both internally and externally that need to be mentioned. We cannot draw any conclusions about effects and causes, because our study is cross-sectional. Second, we depended on self-reported health and chronic diseases because we were unable to obtain access to objective medical histories and records for the participants. Third, CRC screening was assessed by self-report, making measurement biases possible. Fourth, this study did not include non-invasive, fecal-based screening tests which may be as effective as colonoscopy. Despite these drawbacks, the results add to our knowledge of factors that affect whether or not older African Americans in underprivileged areas have CRC screenings. This could help reduce cancer inequalities in such communities.

## Conclusion

 To improve CRC screening rates among African Americans, it is important to gain a deeper understanding of the myriad of factors that play a role. Our findings suggest that the absence of a provider recommendation is the primary barrier preventing underserved African American middle-aged and older adults from obtaining CRC screening. In addition, our data revealed significant associations between some of the predisposing characteristic of participants (gender and living arrangement), possession of HMO membership, satisfaction with access to and quality of medical care, participants’ physical and mental health, and obtaining CRC screening. These findings provide strong support for the notion that disparities in health care for African Americans can be traced back to four main factors: patients, providers, the healthcare system, and society as a whole.^86^ These concepts emphasize the need for establishing multifaceted, community-based, theory-driven, culturally sensitive, and cost-effective CRC screening interventions that recognize and address the diverse constraints to cancer screening experienced by this of population. This research is in line with recent systematic evaluations of cancer screening in minority populations, which have concluded that targeted interventions are required to increase cancer screening rates among individuals from underrepresented racial and ethnic groups.^94^

## Author Contributions

**Conceptualization:** Mohsen Bazargan, Sharon Cobb.

**Data curation: **Edward Adinkrah.

**Funding acquisition: **Mohsen Bazargan.

**Formal Analysis:** Sharon Cobb, Mohsen Bazargan, Edward Adinkrah

**Investigation:** Mohsen Bazargan, Sharon Cobb.

**Project administration: **Edward Adinkrah, Mohsen Bazargan

**Supervision:** Mohsen Bazargan.

**Writing – original draft:** Sharon Cobb, Tavonia Ekwegh, Edward Adinkrah, Hoorolnesa Ameli, Attallah Dillard, Lucy W. Kibe, Mohsen Bazargan.

**Writing – review & editing: **Lucy W. Kibe, Mohsen Bazargan, Tavonia Ekwegh.

## Funding

 This study was supported by the National Institute of Minority Health and Health Disparities under award number U54MD007598. In addition, as scholars of the Clinical Research Education and Career Development (CRECD) program at Charles R. Drew University of Medicine and Science (CDU), Drs. Cobb, Adinkrah, Ekwegh, and Kibe efforts were supported by the NIMHD/NIH Award number R25 MD007610.

## Ethical Approval

 Ethical approval for the study was obtained from the Institutional Review Committee of the CDU (IRB Approval Number1 563 927). Participants provided informed consent. The authors assert that all procedures contributing to this work comply with the ethical standards of the relevant national and institutional committees. All adults who participated in this study were given information about the study, including potential risks and benefits. The consent form significantly described the purpose of the study. Ample opportunity was allowed for participants and their family members to ask questions and discuss what it meant. All participants signed the consent forms. Illiterate participants provided consent in the presence of a reliable or legally authorized family member. The consent form explained that those who participated also gave their consent to utilize the information in a non-identified format for scientific and popular publications.

## Competing Interests

 All authors certify that they have no affiliations with or involvement in any organization or entity with any financial or non-financial interest in the subject matter or materials discussed in this manuscript. There are no any conflicts and financial interests to declare. No connections, direct or indirect, or other situations that might raise the question of bias in work reported for any of authors or for the associated departments or CDU.
